# Clinical Outcomes, Risk Communication, and Follow-Up After Living Kidney Donation: A Retrospective Observational Cohort Study

**DOI:** 10.3390/jcm15207941

**Published:** 2026-10-14

**Authors:** Mahdi Tarabeih, Jamal Qaddumi, Osama Sawalmeh, Sajeda Hamadi

**Affiliations:** 1 Department of Nephrology, Faculty of Nursing, An-Najah National University, Nablus P.O. Box 7, Palestine; 2 Faculty of Medicine, Arab American University, Jenin P.O. Box 240, Palestine; 3 Clinical Research in Health Science, Faculty of Graduate Studies, An-Najah National University, Nablus P.O. Box 7, Palestine

**Keywords:** living kidney donation, donor safety, kidney function, estimated glomerular filtration rate, informed consent, risk communication, post-donation follow-up, cardiometabolic risk

## Abstract

**Background/Objectives**: Living kidney donation expands access to transplantation, but donor safety requires effective risk communication and sustained surveillance. We evaluated renal and cardiometabolic changes after donation in conjunction with donor-reported risk communication and follow-up. **Methods**: This observational cohort study included 91 living kidney donors with paired assessments before donation and 16–18 months after nephrectomy; the analytic cohort included only donors for whom both paired clinical assessments were available. Outcomes included serum creatinine, estimated glomerular filtration rate (eGFR), weight, body mass index (BMI), and blood pressure; counseling and follow-up were assessed retrospectively using a structured questionnaire of donor-reported recall, administered at the post-donation assessment. Changes were summarized as median paired changes with interquartile ranges for non-normally distributed outcomes and as mean paired changes with t-based 95% confidence intervals for normally distributed outcomes; the multiplicity of the six paired comparisons was addressed using a conservative Bonferroni-adjusted significance threshold (0.05/6 = 0.0083). **Results**: Serum creatinine increased by a median of 0.24 mg/dL, and eGFR had a mean paired change of −22.49 mL/min/1.73 m^2^ (95% CI −23.16 to −21.83; t(90) = −67.265; *p* < 0.001). Weight (median +5.0 kg), BMI (median +1.4 kg/m^2^), systolic blood pressure (median +4.0 mmHg), and diastolic blood pressure (mean +5.68 mmHg) also increased (all *p* < 0.001; significant under the Bonferroni threshold). No donor had an eGFR < 60 mL/min/1.73 m^2^ at follow-up. Only 15.4% considered long-term complications adequate, 6.6% recalled counseling regarding future chronic kidney disease or end-stage renal disease, and 19.8% reported regular nephrology follow-up. **Conclusions**: Although reduced eGFR is an expected physiological consequence of nephrectomy, concurrent increases in weight and blood pressure observed during follow-up warrant surveillance. Donor-reported gaps in recall of risk communication and in reported follow-up support standardized informed consent, individualized counseling, verification of understanding, and structured long-term multidisciplinary care to strengthen donor safety and continuity across transplant programs, nephrology, and primary care services; because the design was observational, without a non-donor comparator, and counseling outcomes were based on donor recall, these findings should be interpreted as associations and reported care gaps rather than evidence of causation.

## 1. Introduction

Living kidney donation remains a cornerstone of kidney transplantation because it expands organ availability and generally provides superior recipient outcomes compared with deceased-donor transplantation. Simultaneously, donor nephrectomy is ethically distinctive since la healthy person accepts operative and long-term medical risk without direct therapeutic benefit. Accordingly, donor evaluation and acceptance should be grounded in transparent informed consent, careful risk stratification, and durable post-donation surveillance [1,2,3,4].

Contemporary evidence indicates that donation is followed by predictable physiologic changes rather than complete biological neutrality. Renal function declines immediately after nephrectomy because of a reduced nephron mass, followed by variable compensatory recovery over time. The magnitude of long-term risk appears to be modified by baseline donor characteristics, i.e., age, kidney function, obesity, blood pressure, and metabolic risk, while the absolute risk of kidney failure remains low for most carefully selected donors [5,6,7,8,9,10,11]. Blood pressure trajectories and cardiometabolic drift also deserve attention, since post-donation hypertension and excess adiposity may influence subsequent renal recovery and long-term cardiovascular health [9,12,13,14,15,16,17].

The ethical relevance of these data is direct. If donation may alter renal and cardiovascular risk profiles, the consent process should communicate these issues clearly and be paired with structured long-term follow-up. However, prior research shows marked heterogeneity in informed-consent practice and persistent gaps in donor understanding of long-term risk [18,19,20].

Although previous studies have examined clinical outcomes after living kidney donation [5,21] and others have evaluated informed-consent practices and donor knowledge [22,23], these domains have more commonly been examined separately than concurrently within the same donor cohort. For example, the PRINCE study evaluated living kidney donor knowledge and informed-consent practices in a multicenter cohort, highlighting gaps in donor knowledge regarding long-term complications [22]. Other studies have examined post-donation kidney function and long-term clinical outcomes, including changes in eGFR and other health indicators [5,21]. Integrating paired pre- and post-donation clinical measurements with donor-reported recall of counseling and follow-up experiences may therefore provide a more comprehensive assessment of donor care by examining clinical trajectories alongside donors’ perceptions of risk communication and continuity of care. Therefore, this observational cohort study evaluated paired pre- and post-donation clinical outcomes, together with donor-reported recall of counseling and follow-up among living kidney donors.

## 2. Methods

### 2.1. Study Design and Participants

This observational cohort study included living kidney donors who donated between 2020 and 2023; the most recent donation included in the paired analysis was performed in 2023, ensuring that every included donor had completed the predefined 16–18-month post-donation assessment before the closing of data collection in December 2025. Eligible donors were identified through transplant follow-up records and were included if paired pre-donation and post-donation clinical data were available. A total of 110 donors were screened. Of these, 19 were excluded: 9 were unable to continue study follow-up because they had traveled abroad, and 10 did not complete the required post-donation investigations despite repeated requests. Consequently, 91 donors (82.7% of those screened) comprised the final analytic cohort. All included participants had both pre-donation baseline assessments and corresponding post-donation clinical evaluations performed 16–18 months after donation, allowing within-subject comparisons over time. Baseline characteristics of the 19 excluded donors did not differ systematically from those of the included donors with respect to the variables available for comparison; however, because post-donation follow-up information was, by definition, unavailable for the excluded donors, exclusion of these donors may overestimate the adequacy of post-donation follow-up, and this source of selection bias is acknowledged in Section 4.2.

The clinical component of the study consisted of paired medical-record data obtained at two predefined time points (pre-donation baseline and 16–18 months post donation). Data collection occurred from April 2020 through December 2025 using a combination of online survey distribution and interviewer-administered assessments for participants who preferred in-person participation or did not routinely use the Internet. Clinical information was obtained from paired medical records, whereas donor-reported information regarding informed consent and follow-up experiences was collected retrospectively using a structured questionnaire administered once, at the post-donation assessment; the questionnaire therefore assessed donors’ recall of pre-donation counseling and of post-donation follow-up rather than prospectively documented practice. The study used a paired pre–post design in which each participant served as his or her own control for longitudinal comparisons (see Supplementary Questionnaire S1).

### 2.2. Disclosure Assessment

Disclosure during the informed-consent process was assessed using a study-specific structured questionnaire informed by the PRINCE study and its protocol and aligned with KDIGO and KDOQI recommendations for living kidney donor education and long-term follow-up [1,2,3,18,19,22,23]. The questionnaire assessed donors’ retrospective recall of information provided before donation rather than the documented content of medical records or institutional consent materials.

Donors were asked about counseling concerning postoperative recovery, living with one kidney, compensatory kidney hypertrophy, long-term renal and cardiovascular risks, and recommended post-donation follow-up. The assessed risks included hypertension, proteinuria, declining kidney function, and chronic kidney disease or end-stage renal disease. Female participants were also asked about counseling regarding potential future pregnancy and maternal–fetal outcomes. Because information on parity, reproductive status, and pregnancy intentions at donation was unavailable, pregnancy-counseling responses are reported for all female participants. Participants also reported whether they had continued follow-up with a nephrologist. Because these outcomes were self-reported and descriptive, they are presented as frequencies and percentages without subgroup comparisons or causal inference.

The questionnaire was developed in English and translated into Arabic, with the Arabic version reviewed by a native Arabic speaker for linguistic clarity and cultural appropriateness. Before administration, its content, relevance, comprehensibility, and coverage were qualitatively reviewed by two nephrologists, one kidney transplant surgeon, one transplant coordinator, and one senior transplant-unit nurse. This constituted expert content and face review rather than formal psychometric validation; no formal pilot or reliability testing was performed. “Adequate information regarding long-term complications” was assessed using a single donor-reported item with the response options of “Yes,” “No,” and “I do not know” and therefore reflected perceived rather than objectively verified adequacy. Participants were not asked to distinguish between information communicated, understood, and retained; consequently, the study cannot determine whether information was not provided, was inadequately communicated or understood, or was initially understood but not retained. The complete questionnaire wording and response options are provided in Supplementary Questionnaire S1.

### 2.3. Clinical and Laboratory Variables

Clinical assessments were performed at two predefined time points: immediately pre kidney donation (baseline) and 16–18 months post donation (follow-up). Paired variables included body weight, serum creatinine, estimated glomerular filtration rate (eGFR), body mass index (BMI), systolic blood pressure (SBP), and diastolic blood pressure (DBP). Height, age, sex, education, marital status, religion, and religious observance were recorded as baseline descriptive characteristics.

Following an overnight fast, peripheral venous blood samples were collected by trained nursing staff and analyzed in the hospital’s accredited clinical laboratory using the Cobas^®^ 8000 modular analyzer series (Roche Diagnostics GmbH, Mannheim, Germany) according to standardized quality-control procedures. Serum creatinine was measured as part of the routine clinical laboratory assessment. Laboratory personnel were blinded to the participants’ demographic and clinical characteristics to minimize measurement bias.

eGFR was calculated using the 2021 Chronic Kidney Disease Epidemiology Collaboration (CKD-EPI) race-free creatinine equation and implemented according to National Kidney Foundation laboratory guidance [3,4]: eGFR = 142 × min(Scr/κ,1)^α × max(Scr/κ,1)^−1.200 × 0.9938^Age × 1.012 (if female), where Scr is serum creatinine (mg/dL), κ = 0.7 for females and 0.9 for males, and α = −0.241 for females and −0.302 for males.

Body weight was measured with participants wearing light clothing and no shoes. Standing height was measured using a wall-mounted stadiometer, and BMI was calculated as weight in kilograms divided by the square of height in meters (kg/m^2^). Blood pressure was measured using an automated digital blood pressure monitor (OMRON Healthcare Co., Ltd., Kyoto, Japan) after participants had rested for 15 min in the seated position. Measurements were obtained from the left arm using an appropriately sized cuff; three consecutive blood-pressure readings were obtained, and the mean of the three measurements was recorded as the final SBP and DBP values for analysis. The same standardized measurement protocol was used at both the pre-donation baseline and the 16–18-month post-donation follow-up. The same clinical variables were assessed at both study time points to permit paired longitudinal comparisons.

### 2.4. Statistical Analysis

Statistical analyses were performed using paired comparisons, since all participants underwent both pre- and post-donation assessments. Continuous variables are presented as mean ± standard deviation (SD), whereas categorical variables are summarized as frequencies and percentages.

Questionnaire responses were analyzed descriptively and are reported as frequencies and percentages; no inferential statistical tests were performed. The normality of paired change scores was assessed using the Shapiro–Wilk test. As change scores for serum creatinine, body weight, BMI, and SBP were non-normally distributed, these variables were analyzed using the Wilcoxon signed-rank test and are reported as median paired changes with interquartile ranges (IQRs). Change scores for eGFR and DBP were approximately normally distributed and were therefore analyzed using paired-samples *t* tests, with results reported as mean paired differences, degrees of freedom (df = 90 for 91 complete pairs), and 95% confidence intervals calculated using the standard t-based confidence interval for the mean paired difference. Effect sizes were reported as r for Wilcoxon signed-rank tests and Cohen’s d for paired *t* tests. Because six paired clinical outcomes were tested, a conservative Bonferroni-adjusted significance threshold of 0.05/6 = 0.0083 was applied to account for multiple comparisons. Statistical significance was therefore considered at a two-sided *p* value < 0.0083 after Bonferroni adjustment. All analyses were performed using IBM SPSS Statistics (Version 27; IBM Corp., Armonk, NY, USA). The reported paired-test statistics were cross-checked against the accompanying SPSS output.

### 2.5. Ethics

The study was conducted in accordance with the ethical principles of the Declaration of Helsinki and complied with all applicable institutional and national regulations governing research involving human participants. The study protocol received prospective approval from the Institutional Review Board of An-Najah National University Hospital (IRB Approval No. MTA: 2018015) on 15 April 2020, before the initiation of study data collection. The study subsequently continued according to the approved protocol, with donor follow-up and data collection extending through December 2025. Written informed consent was obtained from all participants prior to study participation. Participant confidentiality was maintained throughout the study. Data were anonymized prior to statistical analysis, and all study information was handled in accordance with institutional requirements for the protection of participant privacy.

## 3. Results

The analytic cohort consisted of 91 living kidney donors. Sixty (65.9%) were male, and 31 (34.1%) were female. Mean age was 37.79 ± 8.07 years. Most donors were aged 30–39 or 40–49, 54.9% had received an academic education, and 76.9% were married. Additional demographic characteristics are summarized in Table 1. Donors were predominantly between 30 and 49 years of age, accounting for 77 of the 91 participants (84.6%).

**Figure 1:**
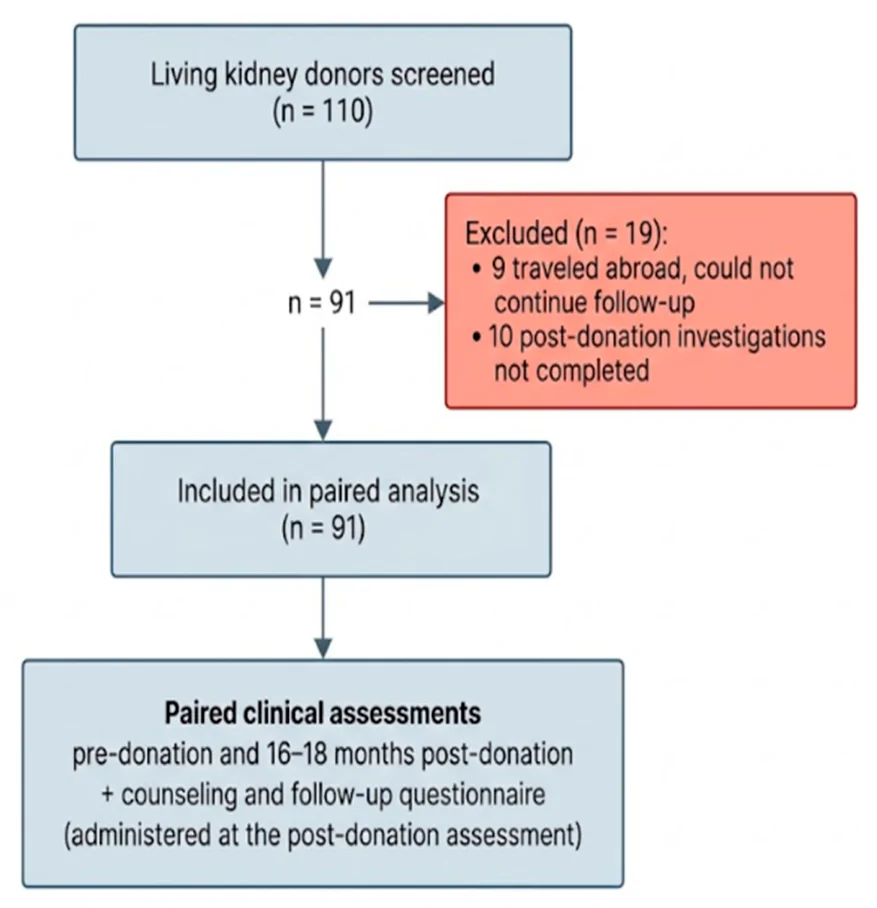
Participant flow: screened, excluded (with reasons), and included donors.

Of the 110 donors initially screened, 91 (82.7%) met the inclusion criteria and were included in the final analysis, whereas 19 (17.3%) were excluded: 9 were unable to continue study follow-up because they had traveled abroad, and 10 did not complete the required post-donation investigations despite repeated requests (Figure 1). Complete paired clinical data were available for all 91 included participants. Questionnaire findings are reported descriptively, and pregnancy-counseling responses are presented for all 31 female participants since information regarding parity and future pregnancy intentions was unavailable.

Baseline and follow-up clinical measurements, together with the paired changes and their statistical significance, are presented in Table 2.

Table 2 summarizes the paired post-donation changes and statistical comparisons. All six evaluated clinical outcomes showed statistically significant changes after donation (all *p* < 0.001). These findings indicate consistent within-participant changes during the 16–18-month follow-up period.

To complement the continuous paired analyses with clinically interpretable outcomes, we additionally evaluated clinically relevant post-donation thresholds. At the 16–18-month assessment, 0 of 91 (0.0%) donors had an eGFR < 60 mL/min/1.73 m^2^ (the lowest observed follow-up eGFR was 83.2 mL/min/1.73 m^2^). Individual-level classification of incident hypertension, overweight/obesity categories, and clinically relevant weight gain could not be reliably derived from the available aggregate paired measurements and was therefore not reported; this is acknowledged in Section 4.2, and clinically defined categorical outcomes should be evaluated in future studies.

Overall, the findings demonstrated low donor-reported recall of several components of pre-donation counseling and risk communication, together with limited self-reported post-donation follow-up. Only 23 donors (25.3%) reported being informed as to which kidney was planned for donation, whereas 64 donors (70.3%) indicated that this information had not been discussed. Similarly, only 27 donors (29.7%) recalled receiving information as to the medical considerations involved in selecting the kidney for donation.

Knowledge regarding physiological adaptation after nephrectomy was limited. Only 14 donors (15.4%) reported being informed that the remaining kidney may undergo compensatory hypertrophy and functional adaptation following donation, whereas 71 donors (78.0%) denied receiving such information. Awareness of the implications of living with a single kidney was also low, with only 16 donors (17.6%) reporting adequate awareness prior to donation, whereas nearly one-third of the participants (31.9%) were uncertain concerning this issue.

Regarding long-term renal outcomes, only 14 donors (15.4%) recalled counseling about the risk of post-donation hypertension, 9 donors (9.9%) reported receiving information regarding proteinuria, and only 6 donors (6.6%) recalled discussion of the potential risk of chronic kidney disease or end-stage renal disease. Although 28 donors (30.8%) reported being informed that kidney function may decline over time after donation, more than two-thirds of the participants either denied receiving such information or were uncertain about it. Only 14 donors (15.4%) considered the information provided regarding long-term complications of kidney donation to be adequate, whereas 60 donors (65.9%) considered it insufficient. These findings represent donor-reported recall and perceived adequacy of counseling rather than an objective assessment of the provided information.

Post-donation surveillance was similarly limited. Only 18 donors (19.8%) reported regular nephrology follow-up post-donation, and only 15 donors (16.5%) continued with a regular follow-up with a primary care physician. Furthermore, only 12 donors (13.2%) reported ongoing communication with transplant coordinators following donation.

Among the 31 female donors, 2 (6.5%) recalled receiving counseling regarding the potential implications of kidney donation for future pregnancy and maternal–fetal outcomes. However, information regarding parity, reproductive potential, and future pregnancy intentions at the time of donation was unavailable. Therefore, this finding is presented descriptively for the entire female subgroup and should not be interpreted as indicating inadequate pregnancy counseling among all female donors or among women who were specifically considering future pregnancy.

## 4. Discussion

This study integrates clinical and ethical dimensions of living kidney donor care within a single cohort. Three main findings deserve emphasis. First, donors experienced a significant increase in serum creatinine and a significant decline in eGFR at 16–18 months after donation. Second, weight, BMI, SBP, and DBP also increased significantly. Third, donor-reported recall of counseling and reported specialist follow-up were limited despite clinically meaningful changes observed after donation.

The mean post-donation eGFR in this cohort was approximately 85 mL/min/1.73 m^2^. Although a reduction in eGFR is expected after living donor nephrectomy because of the reduction in renal mass, the remaining kidney undergoes compensatory functional changes, and post-donation kidney function may continue to change over time [21]. Therefore, the observed post-donation eGFR should not be interpreted in isolation as evidence for or against kidney disease or future clinical risk. The magnitude of the decline observed in this cohort underscores the importance of interpreting renal function trajectories rather than relying solely on absolute eGFR values. Longitudinal assessment may help identify donors who require closer clinical surveillance.

Beyond facilitating the early identification of changes in renal function, structured follow-up may provide opportunities to reinforce healthy lifestyle behaviors and address emerging cardiovascular or metabolic risk factors. It may also allow healthcare professionals to provide appropriate counseling and reassurance to donors regarding their long-term health.

The renal findings are consistent with the established physiologic consequences of unilateral nephrectomy. In the present cohort, eGFR declined by approximately 22.5 mL/min/1.73 m^2^; serum creatinine increased by a mean of approximately 0.23 mg/dL. These results align with studies showing that post-donation renal function declines initially and then follows heterogeneous recovery trajectories influenced by donor baseline characteristics and early post-donation kidney function [5,6,7,8,9,10,11,14].

Our findings also support continued use of standardized eGFR reporting methods and transparent explanation of their clinical meaning during donor counseling [24,25,26]. The observed reduction in eGFR should not automatically be interpreted as pathological renal deterioration, because reduced filtration is an expected physiological consequence of unilateral nephrectomy. Nevertheless, the magnitude and individual trajectory of this reduction remain clinically relevant, particularly when accompanied by progressive decline, hypertension, diabetes, albuminuria, or excessive weight gain.

The exceptionally large standardized effect size for eGFR should be interpreted cautiously. eGFR was mathematically derived from serum creatinine, age, and sex and is therefore not statistically independent of serum creatinine. In addition, the narrow variation in follow-up eGFR values may have increased the standardized effect estimate. Absolute changes and their clinical context may therefore be more informative than the standardized effect size alone. The observed increase in body weight and BMI may have important long-term implications since excess adiposity is a recognized risk factor for hypertension, chronic kidney disease progression, and adverse cardiovascular outcomes. Although causality cannot be inferred from the present study, these findings support integrating dietary counseling, regular physical activity, and weight-management strategies into routine post-donation care.

Blood pressure and anthropometric changes were also notable. The simultaneous rise in BMI, SBP, and DBP suggests a cardiometabolic drift during follow-up, a pattern that is increasingly recognized in the donor literature. Meta-analytic and cohort data indicate that obesity, male sex, and longer follow-up are associated with less favorable blood pressure and renal outcomes after donation [9,12,13,15,16]. These observations do not negate the overall value of living donation, but they do reinforce the need for long-term lifestyle counseling, blood pressure surveillance, and individualized risk communication before and after nephrectomy. It must be emphasized that the changes described here are changes observed after donation and should not be interpreted as changes caused by donation: in the absence of a matched non-donor comparator, the observed increases in weight and blood pressure cannot be attributed exclusively to kidney donation, since diet, physical activity, smoking, medication use, psychosocial stress, incident diabetes, family history, aging, secular trends, and other socioeconomic or clinical factors were not assessed and may have contributed to the observed changes.

The disclosure findings are ethically important. Only a small minority of donors recalled counseling regarding life with one kidney or major long-term risks. It should also be acknowledged that donor recall may not perfectly reflect the information originally provided during the consent process. Nevertheless, donor understanding and retention of information are fundamental components of valid informed consent, making donor-reported recall clinically and ethically relevant, irrespective of the completeness of the original counseling. Prior work has documented heterogeneity in informed-consent practice, suboptimal donor knowledge, and the need for structured, staged approaches to communication [1,18,19].

In this context, low donor-reported recall of important risk information is clinically and ethically relevant because adequate understanding and retention of information are fundamental components of informed consent. However, because counseling was assessed retrospectively through donor recall and was not verified against contemporaneous documentation, these findings should not be interpreted as direct evidence that the information was not originally provided; recall nevertheless weakens the practical autonomy of the donor when it is low, because it becomes difficult for donors to interpret post-donation symptoms and laboratory changes. Programs should therefore use standardized consent pathways, reinforce long-term risk information at multiple time points, and verify donor understanding rather than relying on one-time verbal explanations. Because counseling findings were based on retrospective donor recall and were not verified against consent documents, educational materials, or clinical records, the study cannot distinguish between information that was not provided, information that was communicated inadequately, and information that was provided but not retained.

The findings presented in Table 3 further highlight low donor-reported recall of several components of donor education and informed consent. Despite undergoing formal donor evaluation, only a minority of participants recalled receiving information regarding compensatory renal adaptation, hypertension, proteinuria, long-term decline in kidney function, or the future risk of chronic kidney disease and end-stage renal disease. Similar findings have been reported internationally. The multicenter PRINCE study demonstrated that donor knowledge regarding long-term consequences of kidney donation remained suboptimal even after completion of the donor workup process, with long-term risks representing the domain with the poorest understanding among donors [22].

Likewise, Rodrigue et al. [27] reported substantial variability among transplant centers in the content and delivery of informed consent, particularly concerning long-term medical risks and post-donation expectations. Furthermore, the low rates of post-donation nephrology follow-up and transplant coordinator contact observed in our cohort contrast with current international recommendations advocating lifelong surveillance of living kidney donors, including periodic monitoring of kidney function, blood pressure, and cardiovascular risk factors [3]. Collectively, these findings suggest that standardized educational pathways, repeated reinforcement of key messages, and structured long-term follow-up programs are necessary to optimize donor safety and support truly informed decision-making.

The low rate of regular nephrology follow-up reported in this cohort is concerning. Contemporary guidance emphasizes that donor care should extend well beyond the perioperative period, with continued monitoring of kidney function, blood pressure, metabolic risk factors, and pregnancy-related issues when relevant [3,4,18]. A clearly defined transition-of-care pathway should be established before donors leave the transplant program. The transplant center should provide each donor and the primary care physician with an individualized surveillance plan, specify the required frequency of laboratory and clinical assessments, define criteria for nephrology referral, and establish procedures for contacting donors who miss scheduled follow-up visits.

Taken together, these findings indicate that donor care should not end with a successful nephrectomy or favorable recipient outcomes. Post-donation renal adaptation should be interpreted within its expected physiological context, while concurrent weight gain, increasing blood pressure, limited recall of long-term risks, and low follow-up attendance should be regarded as potentially modifiable care gaps. However, the observational design, short follow-up period, absence of a non-donor comparator, and reliance on donor recall limit causal interpretation. The findings therefore support stronger donor surveillance while also underscoring the need for prospective controlled studies.

Future multicenter prospective studies with a longer follow-up are needed to determine whether the observed changes in renal function, blood pressure, and body weight persist, stabilize, or progress over time. Incorporating measurements such as albuminuria, cardiovascular outcomes, quality of life, and patient-reported experiences would provide a more comprehensive understanding of long-term donor health and help refine individualized risk prediction models.

### 4.1. Recommendations

The present findings indicate low donor-reported recall of several aspects of pre-donation counseling and limited self-reported post-donation follow-up. These findings support the importance of ensuring that donor education is understandable, appropriately documented, and reinforced over time. However, because this study assessed retrospective donor recall rather than the actual content of the informed-consent process, the findings should not be interpreted as direct evidence of inadequate disclosure.

Consistent with existing living-donor guidelines and recommendations [3,4,18], transplant programs should promote structured pre-donation counseling and continued post-donation clinical surveillance. The broader practice measures proposed by the authors individualized pre-donation risk assessment, written post-donation care plans, structured follow-up intervals with kidney-function and blood-pressure monitoring, lifestyle and pregnancy counseling where relevant, active recall systems for donors lost to follow-up, national donor registries, and standardized quality indicators are recommendations for future implementation and evaluation rather than interventions demonstrated to be effective by the present study. Future prospective studies should evaluate counseling using contemporaneous documentation and assess longer-term clinical outcomes and follow-up adherence.

### 4.2. Limitations

This study has several important limitations. Selection bias is possible due to the fact that 19 of the 110 screened donors were excluded owing to unavailable post-donation clinical data (9 had traveled abroad and 10 did not complete the required post-donation investigations). Lack of regular follow-up contributed to their exclusion and the study may therefore overestimate the adequacy of post-donation surveillance; the absence of follow-up was itself an outcome of interest, so this exclusion may substantially bias the reported estimates of follow-up adequacy. The absence of a matched non-donor comparison group prevents determination of whether changes in weight, blood pressure, and other cardiometabolic outcomes exceeded changes that might have occurred naturally over the same period in similar adults who did not donate a kidney; accordingly, the observed cardiometabolic changes should be understood as changes observed after donation rather than changes caused by donation. Only limited baseline variables (age, sex, height, weight, BMI, serum creatinine, eGFR, SBP, and DBP) were available, and a detailed comparison of the baseline characteristics of the 19 excluded donors with those of the 91 included donors was not possible from the available data.

The follow-up period of 16–18 months captures early to intermediate post-donation changes; however, it is insufficient to characterize long-term renal trajectories, cardiovascular events, pregnancy outcomes, kidney failure, or mortality. Individual follow-up durations varied within this predefined interval, and the exact mean and range of follow-up times could not be reported from the available data. Informed-consent and follow-up outcomes were based on donor recall and were not verified against consent forms, educational materials, clinician notes, or transplant-program documentation; the study therefore cannot establish that counseling was not provided, only that participants did not recall receiving or understanding particular information. The number of donors with missing questionnaire responses was minimal and comparable across items. Albuminuria, quantified proteinuria, cystatin C, and measured GFR were unavailable, and renal assessment therefore relied mainly on serum creatinine-based eGFR. Potential confounders such as smoking, diet, physical activity, family history, medication use, socioeconomic status, baseline metabolic status, incident diabetes, and psychosocial stress were not comprehensively assessed; the absence of these variables constitutes a major limitation that may account for part of the observed changes in weight and blood pressure. The modest sample size and limited recruitment context restrict generalizability and precluded robust subgroup or multivariable analyses. No adjustment for multiple comparisons was prespecified in the original analysis plan; the Bonferroni-adjusted threshold applied in the revised analysis is reported in Section 2.4. In addition, information regarding parity, reproductive intentions, and actual pregnancy outcomes was unavailable, limiting interpretation of the pregnancy-counseling findings. Finally, the exceptionally narrow variability observed for follow-up eGFR (SD 1.39 mL/min/1.73 m^2^; observed range 83.2–89.1 mL/min/1.73 m^2^) and the very large standardized effect estimate warrant cautious interpretation because eGFR was derived mathematically from serum creatinine rather than measured directly. Despite these limitations, the paired longitudinal design and integration of objective clinical outcomes with donor-reported experiences provide useful insight into potentially modifiable gaps in living kidney donor care.

## 5. Conclusions

Living kidney donors in this cohort demonstrated significant changes in renal function, body weight, BMI, and blood pressure during the 16–18 months following donation. The reduction in eGFR was consistent with the expected physiological consequences of unilateral nephrectomy, whereas concurrent increases in weight and blood pressure highlight the importance of continued cardiometabolic surveillance. Donor-reported understanding of long-term risks was limited, and regular nephrology follow-up was reported by only 18 participants (19.8%).

These findings support the implementation of standardized and repeated informed-consent procedures; individualized risk communication; and clearly coordinated long-term follow-up involving transplant programs, nephrologists, and primary care clinicians. However, because this was a retrospective study without a non-donor control group and counseling outcomes were based on donor recall, the findings should be interpreted as evidence of important associations, observed post-donation changes, and care gaps rather than proof that donation alone caused all observed clinical changes or that counseling was definitively inadequate. Larger prospective multicenter studies with longer follow-up, albuminuria assessment, and clinically defined outcome categories are needed to confirm these findings and inform donor-care policy.

## Figures and Tables

**Table 1:** Demographic characteristics of the study participants (N = 91).

Characteristic	N	%
**Gender**		
Male	60	65.9
Female	31	34.1
**Age at donation (years)**		
18–29	18	19.8
30–39	37	40.7
40–49	36	39.6
**Education**		
Up to 12 years	41	45.1
Academic	50	54.9
**Marital status**		
Single/Divorced	21	23.1
Married	70	76.9
**Religion**		
Muslim	13	14.3
Christian	5	5.5
Jewish	73	80.2
**Religious observance**		
Secular	8	8.8
Atheist	14	15.4
Traditional	32	35.2
Ultra-Orthodox	37	40.7

**Table 2:** Paired pre- and post-donation clinical measurements and paired changes after donation (n = 91 complete pairs).

Variable	Pre-Donation (Mean ± SD)	Post-Donation (Mean ± SD)	Change (Post − Pre)	*p*-Value
Age, years	37.79 ± 8.07	—	—	—
Height, m	1.76 ± 0.05	—	—	—
Weight, kg	74.42 ± 5.62	80.40 ± 5.59	Median +5.0 kg (IQR 4.0–8.0)	<0.001
Serum creatinine, mg/dL	0.78 ± 0.07	1.02 ± 0.07	Median +0.24 mg/dL (IQR 0.23–0.24)	<0.001
eGFR, mL/min/1.73 m^2^	107.65 ± 3.87	85.16 ± 1.39	Mean −22.49 (95% CI −23.16 to −21.83)	<0.001
BMI, kg/m^2^	24.88 ± 1.05	26.36 ± 0.75	Median +1.4 kg/m^2^ (IQR 1.0–2.0)	<0.001
SBP, mmHg	117.58 ± 3.72	122.92 ± 1.78	Median +4.0 mmHg (IQR 2.0–8.0)	<0.001
DBP, mmHg	71.74 ± 4.22	77.42 ± 3.96	Mean +5.68 mmHg (95% CI 4.99–6.37)	<0.001

Note: Values are presented as mean ± SD unless otherwise indicated. Changes are post-donation minus pre-donation and are reported as median (IQR) for non-normally distributed variables and as mean (95% CI) for normally distributed variables. The reported *p*-values are unadjusted and all remain below the Bonferroni threshold of 0.0083. Test statistics and effect sizes: weight, Wilcoxon Z = 8.285, r = 0.87; serum creatinine, Wilcoxon Z = 8.296, r = 0.87; BMI, Wilcoxon Z = 8.103, r = 0.85; SBP, Wilcoxon Z = 8.158, r = 0.86; eGFR, t(90) = −67.265, d = 7.05; DBP, t(90) = +16.376, d = 1.72. eGFR, estimated glomerular filtration rate; BMI, body mass index; SBP, systolic blood pressure; DBP, diastolic blood pressure.

**Table 3:** Living kidney donor questionnaire responses (n = 91).

Question	Yes, n (%)	No, n (%)	I Don’t Know, n (%)
Informed which kidney (right or left) was planned for donation	23 (25.3%)	64 (70.3%)	4 (4.4%)
Informed about medical considerations for kidney selection	27 (29.7%)	55 (60.4%)	9 (9.9%)
Informed about compensatory hypertrophy and adaptation of the remaining kidney	14 (15.4%)	71 (78.0%)	6 (6.6%)
Aware of health implications of living with a single kidney	16 (17.6%)	46 (50.5%)	29 (31.9%)
Informed about the risk of hypertension after donation	14 (15.4%)	73 (80.2%)	4 (4.4%)
Informed about the risk of proteinuria after donation	9 (9.9%)	76 (83.5%)	6 (6.6%)
Informed about possible decline in kidney function over time	28 (30.8%)	36 (39.6%)	27 (29.7%)
Informed about possible future CKD or ESRD risk	6 (6.6%)	73 (80.2%)	12 (13.2%)
Received adequate information regarding long-term complications	14 (15.4%)	60 (65.9%)	17 (18.7%)
Continued regular follow-up with a nephrologist	18 (19.8%)	73 (80.2%)	0 (0.0%)
Continued regular follow-up with a primary care physician	15 (16.5%)	76 (83.5%)	0 (0.0%)
Continued contact with the transplant coordinator after donation	12 (13.2%)	73 (80.2%)	6 (6.6%)
**Female participants only (n = 31)**			
Counseled regarding future pregnancy and maternal-fetal outcomes	2 (6.5%)	29 (93.5%)	0 (0.0%)

## Data Availability

The datasets generated and/or analyzed during the current study are not publicly available due to ethical and privacy restrictions but are available from the corresponding author upon reasonable request and subject to approval by the Institutional Review Board.

## Supplementary Materials

The following supporting information can be downloaded at: https://www.mdpi.com/article/10.3390/jcm15207941/s1, Questionnaire S1: Living Kidney Donor Characteristics Clinical Assessment and Recall of Counseling and Follow Up. Table S1: Participant Characteristics. Table S2: Clinical Record Abstraction Form. Table S3: Donor Reported Counseling and Follow Up Questionnaire.

## Abbreviations

The following abbreviations are used in this manuscript:

BMI	Body Mass Index
CI	Confidence Interval
CKD	Chronic Kidney Disease
CKD-EPI	Chronic Kidney Disease Epidemiology Collaboration
DBP	Diastolic Blood Pressure
eGFR	Estimated Glomerular Filtration Rate
ESRD	End-Stage Renal Disease
IRB	Institutional Review Board
KDIGO	Kidney Disease: Improving Global Outcomes
SBP	Systolic Blood Pressure
SD	Standard Deviation
