# Supplementary material for: Clinical Outcomes, Risk Communication, and Follow-Up After Living Kidney Donation: A Retrospective Observational Cohort Study
Source: J Clin Med. 2026 Oct 14;15(20):7941. doi: 10.3390/jcm15207941 (PMC13705969; doi:10.3390/jcm15207941)
Supplement: Supplementary file 1 [file jcm-15-07941-s001.zip › jcm-4533276-supplementary.pdf]

# Supplementary Questionnaire S1

## Living Kidney Donor Characteristics Clinical Assessment and Recall of Counseling and Follow Up

**Purpose and administration.** This study-specific, literature-informed instrument was administered once at the 16–18-month post-donation assessment. Tables S1 and S2 document baseline characteristics and paired clinical information abstracted from medical records. Table S3 records participants' retrospective recall of pre-donation counseling and their reported post-donation follow-up.

**Questionnaire development.** The counseling and follow-up domains were informed by the PRINCE study and its protocol and aligned with KDIGO and KDOQI recommendations. The English questionnaire was translated into Arabic and linguistically reviewed by a native Arabic speaker. Five transplantation professionals—two nephrologists, one kidney transplant surgeon, one transplant coordinator, and one senior transplant-unit nurse—conducted qualitative content and face review. No formal pilot study, psychometric validation, internal-consistency assessment, or test–retest reliability assessment was performed.

**Interpretation.** Responses reflect donor-reported recall and perceived adequacy; they do not independently verify the content of consent forms, educational materials, medical records, or clinician documentation. Participants were not asked to distinguish information communicated from information understood or retained.

### Table S1 Participant Characteristics

**Source:** Participant report or study record, as applicable. Select one response unless otherwise indicated.

| Field | Response categories | Recorded response |
|---|---|---|
| Study participant ID | De-identified study code | ________________ |
| Sex | Male; Female | ________________ |
| Age at donation | Age in completed years | _______ years |
| Age category | 18–29; 30–39; 40–49 years | ________________ |
| Education | Up to 12 years; Academic education | ________________ |
| Marital status | Single/Divorced; Married | ________________ |
| Religion | Muslim; Christian; Jewish | ________________ |
| Religious observance | Secular; Atheist; Traditional; Ultra-Orthodox | ________________ |

### Table S2 Clinical Record Abstraction Form

**Source:** Paired medical-record assessments immediately before donation and 16–18 months after nephrectomy. These variables were not questionnaire-based.

| Clinical variable | Unit | Pre-donation | 16–18 months post-donation |
|---|---|---|---|
| Assessment date | DD/MM/YYYY | ____________ | ____________ |
| Age | years | ____________ | Not applicable |
| Height | m | ____________ | Not applicable |
| Body weight | kg | ____________ | ____________ |
| Serum creatinine | mg/dL | ____________ | ____________ |
| Estimated glomerular filtration rate (eGFR) | mL/min/1.73 m² | ____________ | ____________ |
| Body mass index (BMI) | kg/m² | ____________ | ____________ |
| Systolic blood pressure (SBP) | mmHg | ____________ | ____________ |
| Diastolic blood pressure (DBP) | mmHg | ____________ | ____________ |

## Table S3 Donor Reported Counseling and Follow Up Questionnaire

**Instructions to participants:** Please select one response for each item based on what you recall. Select “I do not know” if you are uncertain or do not remember.

| Item | Question | Yes | No | I do not know |
|---|---|---|---|---|
| 1 | Before donation, were you informed which kidney, right or left, was planned for donation? | ☐ | ☐ | ☐ |
| 2 | Before donation, were you informed about the medical considerations used to determine which kidney would be donated? | ☐ | ☐ | ☐ |
| 3 | Before donation, were you informed that the remaining kidney may undergo compensatory enlargement and functional adaptation? | ☐ | ☐ | ☐ |
| 4 | Before donation, were you made aware of the potential health implications of living with one kidney? | ☐ | ☐ | ☐ |
| 5 | Before donation, were you informed about the possible risk of developing hypertension after kidney donation? | ☐ | ☐ | ☐ |
| 6 | Before donation, were you informed about the possible risk of developing proteinuria after kidney donation? | ☐ | ☐ | ☐ |
| 7 | Before donation, were you informed that your kidney function might decline over time following donation? | ☐ | ☐ | ☐ |
| 8 | Before donation, were you informed about the possible future risk of chronic kidney disease or end-stage renal disease? | ☐ | ☐ | ☐ |
| 9 | In your opinion, did you receive adequate information about the possible long-term complications of kidney donation? | ☐ | ☐ | ☐ |
| 10 | Following donation, did you continue regular follow-up with a nephrologist? | ☐ | ☐ | ☐ |
| 11 | Following donation, did you continue regular follow-up with a primary care physician? | ☐ | ☐ | ☐ |
| 12 | Following donation, did you remain in contact with the transplant coordinator? | ☐ | ☐ | ☐ |
| 13 | For female participants only: Before donation, were you counseled regarding the potential implications of kidney donation for a future pregnancy and maternal–fetal outcomes? | ☐ | ☐ | ☐ |

**Operational definition of Item 9.** A “Yes” response was classified as donor-reported perceived adequacy of information. No numerical cutoff, knowledge score, external criterion, or independent clinical assessment was used. “No” and “I do not know” responses were reported separately.

**Administration record.** Date completed: ____________________ Language: ☐ English ☐ Arabic Study staff initials: __________

**Abbreviations:** BMI, body mass index; CKD, chronic kidney disease; DBP, diastolic blood pressure; eGFR, estimated glomerular filtration rate; ESRD, end-stage renal disease; KDIGO, Kidney Disease: Improving Global Outcomes; KDOQI, Kidney Disease Outcomes Quality Initiative; SBP, systolic blood pressure.
